# BMP-7 Upregulates Id2 Through the MAPK Signaling Pathway to Improve Diabetic Tubulointerstitial Fibrosis and the Intervention of Oxymatrine

**DOI:** 10.3389/fphar.2022.900346

**Published:** 2022-06-02

**Authors:** Yawen Xiao, Dan Liang, Zhiyang Li, Zhaowei Feng, Zhiping Yuan, Fan Zhang, Yuanyuan Wang, Yuxia Zhou, Mingjun Shi, Lingling Liu, Ying Xiao, Bing Guo

**Affiliations:** ^1^ Guizhou Provincial Key Laboratory of Pathogenesis and Drug Research on Common Chronic Diseases, Guizhou Medical University, Guizhou, China; ^2^ Department of Pathophysiology, Guizhou Medical University, Guizhou, China; ^3^ School Hospital, Guizhou Medical University, Guiyang, China

**Keywords:** BMP-7, Id2, MAPKs, oxymatrine, tubulointerstitial fibrosis

## Abstract

Diabetic kidney disease is one of the most serious microvascular complications of diabetes. It progresses irreversibly to end-stage renal disease if left untreated. Bone morphogenetic protein (BMP)-7 is a negative regulator of organ fibrosis and may also play an essential role in tubulointerstitial fibrosis. This study aimed to investigate the precise role and potential molecular mechanisms of BMP-7 in the progression of diabetic nephropathy. In this study, BMP-7 was overexpressed *in vivo* after the replication of the diabetic rat model using streptozotocin. The results showed that BMP-7 inhibited the phosphorylation of related mitogen-activated protein kinase (MAPK) pathways while upregulating the inhibitor of differentiation (Id2) expression and effectively ameliorated pathological renal injury. Further *in vitro* validation showed that the inhibition of the phosphorylation of MAPKs at a high glucose concentration in renal tubular epithelial cells was followed by the upregulation of Id2 protein expression, suggesting that BMP-7 could improve diabetic nephropathy by upregulating Id2 protein levels through the BMP-7–MAPK signaling pathway. Previous laboratory studies found that oxymatrine improved renal fibrotic lesions. However, the exact mechanism is unclear. The present study showed that oxymatrine treatment in a diabetic rat model upregulated BMP-7 protein expression and inhibited MAPK pathway protein phosphorylation levels. These results suggested that oxymatrine improved the epithelial-to-mesenchymal transition process in the early stage of diabetic kidney disease by regulating the BMP-7–MAPK pathway and ameliorated renal tubulointerstitial fibrosis.

## Introduction

Diabetic kidney disease (DKD) is one of the most common and severe chronic microvascular complications of diabetes mellitus (DM). Tubulointerstitial fibrosis (TIF) is considered to be the “final common pathway” to renal function loss in DKD. Renal function and prognosis in patients with DKD may ultimately be more related to TIF than to classical and early glomerular changes (Bonner et al., 2020). Increasing evidence shows that the epithelial-to-mesenchymal transition (EMT) plays a vital role in the TIF process (Liu et al., 2019). Renal fibrosis involves the deposition of the fibrotic matrix and scarring in response to severe or persistent injury. Despite its involvement in the wound healing process, persistent fibrosis can damage tissue structure and organ function, eventually leading to renal failure. Chronic injury to the kidney promotes multiple pathological changes, including EMT, which exerts its pro-fibrotic function by secreting collagen I, III, and V and fibronectin, leading to the accumulation of extracellular matrix (ECM) and, ultimately, tubulointerstitial fibrosis (Yuqing et al., 2021). Many genes and proteins are known to be involved in this complex and influential process of EMT. However, the specific regulatory mechanisms remain unclear, especially regarding the changes in the expression of negative regulators that may play an equally important role in the fibrosis process and their regulatory mechanisms.

Bone morphogenetic proteins (BMPs) are homodimeric members of the transforming growth factor β (TGF-β) superfamily (Aluganti Narasimhulu and Singla, 2021). Kidneys are considered the primary site of BMP-7 synthesis during embryonic and postnatal development (Mo et al., 2021). BMP-7 is an essential regulator for maintaining the typical phenotype of renal tubular epithelial cells. The level of BMP-7 is significantly reduced while increased levels of BMP-7 expression can inhibit EMT in various animal models of acute kidney injury or chronic renal fibrosis (Mo et al., 2021). During renal fibrosis, the effect of BMP-7 to impede the development of fibrosis involves reversing glomerular hypertrophy, reducing tubular atrophy, maintaining the tubular epithelial cell phenotype, delaying the EMT process, and inhibiting ECM synthesis (Sun et al., 2017). As the HDAC inhibitor, SFN prevents diabetes-induced renal fibrosis through epigenetic upregulation of BMP-7 (Kong et al., 2021). BMP-7 effectively inhibits TGF-β1-induced EMT by inhibiting Wnt3/β-linked protein and TGF-β1/Smad2/3 signaling pathways (Song et al., 2020). The collagen formation mediated by TGF-β can be inhibited by BMP-7, and this inhibition is accomplished by BMP-7-induced expression of Inhibitor of differentiation 2 (Id2) in murine pulmonary myofibers. Id2 can bind to the type I collagen A2 promoter to form a heterodimer, rendering it inactive transcriptionally (Kim et al., 2015). Id2 is a negative regulatory protein of nuclear transcription factors widely present in mammalian cells. It is a member of the helix-loop-helix family of transcription factors (Ito et al., 2021). Recent studies have shown that reduced Id2 protein expression plays a vital role in developing fibrosis in various organ tissues. Id2 overexpression after myocardial infarction was found to inhibit cardiac fibrosis (Yin et al., 2019). The overexpression of Id2 maintains the alveolar epithelial cell phenotype in pulmonary fibrosis, attenuating pulmonary fibrosis (Yang et al., 2015). A previous study by our group (Xiao et al., 2019) suggested that the EMT process in renal tubular epithelial cells might be associated with the decrease in Id2 expression. The addition of different doses of BMP-7 (100 and 200 ng/ml) to renal tubular epithelial cells cultured in a high-glucose environment *in vitro* showed that BMP-7 induced the upregulation of Id2 expression in renal tubular epithelial cells cultured in a high concentration of glucose, restored E-cadherin expression, and delayed EMT in renal tubular epithelial cells (Liu et al., 2016), suggesting that BMP-7 regulated Id2 expression in renal tubular epithelial cells under the high-glucose status. However, how BMP-7 regulates Id2 expression in renal tubular epithelial cells is not well understood.

Mitogen-activated protein kinases (MAPKs) regulate various cellular programs, including embryogenesis, proliferation, differentiation, and apoptosis, based on cues from the cell surface, metabolic state, and cellular environment. The MAPK pathway is activated in several glomerular and tubulointerstitial diseases, including diabetic nephropathy; together, they mediate signaling in fibrosis. The inhibition of the MAPK signaling pathway reverses EMT progression and renal fibrosis (Yuqing et al., 2021). Many studies have shown that the MAPK pathway regulates EMT as a non-Smad signaling pathway. The inhibition of the MAPK signaling pathway reversed EMT progression and renal fibrosis (Li et al., 2017; Geng et al., 2020). However, most studies focused on the regulation of these proteins at a single site. Whether BMP-7 regulates Id2 through an MAPK signaling pathway during the transdifferentiation of diabetic renal tubular epithelial cells to mesenchymal cells and the development of renal tubular–interstitial fibrosis has not been reported.

Oxymatrine (OMT) is a quinacrine alkaloid extracted from *Scutellaria baicalensis* with an extensive source and a molecular structure of C_15_H_24_N_2_O_2_ and a molecular weight of 264.360 g/mol. It is confirmed that the protective mechanism of OMT is mainly related to anti-inflammatory, anti-oxidative stress, anti- or pro-apoptotic, antifibrotic, metabolic modulation, and anti-nociception effects *in vitro* and *in vivo*. In addition, OMT can affect various signaling pathways, cells, and cytokines, and maximum therapeutic results can be achieved through these combined effects (Lan et al., 2020). Pre-laboratory studies found that OMT could delay the process of high glucose–induced EMT in renal tubular epithelial cells; the possible mechanism was to facilitate DKD tubulointerstitial fibrosis by inhibiting the transcriptional activation of Twist protein on downstream EMT-related target genes through the upregulation of Id2 expression (Ying et al., 2020). However, the specific mechanism of OMT for Id2 regulation remains to be further investigated.

In summary, BMP-7 has a negative role in regulating EMT in renal tubular epithelial cells in diabetic nephropathy. Still, the specific mechanism of BMP-7 regulation of EMT needs to be studied in depth. Another study confirmed the protective effect of oxymatrine on high glucose–induced renal tubular epithelial cell injury. Based on the aforementioned theories and previous studies, this study applied a holistic animal and cellular model, employing a type I diabetic rat model and cultured renal tubular epithelial cells (NRK-52E) with high glucose as the study subjects, and used gene therapy with the overexpression of BMP-7 adeno-associated virus, OMT, MAPK pathway inhibitors, and other multi-method interventions to investigate the effects of BMP-7 on downstream fibrogenic-related signaling pathways and OMT. We investigated the effect of BMP-7 on downstream fibrogenic signaling pathways and the possible regulatory mechanism of OMT on early DKD–related pathophysiological processes.

## Materials and Method

### Experimental Subjects

Sprague–Dawley (SD) rats were purchased from Beijing Huafukang Biotechnology Co., Ltd. and weighed 160 ± 20 g. The rats were randomly divided into normal control group (NC group), diabetes mellitus model group (DM group), DM + overexpressed BMP-7 group (rAAV + BMP-7 group), and oxymatrine intervention group (OMT group). The type 1 diabetes model was replicated using a single streptozotocin (STZ) (concentration of 55 mg/kg) tail vein injection and randomly divided into NC and DM groups. The model was considered successful with blood glucose greater than or equal to 16.7 mmo/L and maintained stable. The adeno-associated virus overexpressing BMP-7 (rAAV + BMP-7) was given by tail vein injection in the sixth week of model formation as the rAAV + BMP-7 group. The adeno-associated virus (dissolved in sterile saline) was injected intravenously at a dose of 1.5 × 10^12^/animal and continuously fed for 8 weeks before death. The rats in the DM model were treated with OMT starting in the sixth week as the OMT group and injected intraperitoneally (120 mg/kg per rat) daily for 8 weeks before death. Blood and urine samples were collected and tested for relevant biochemical parameters in rats using biochemical assay kits. The sample size for statistical analysis of each independent group was 6 (n = 6).

**FIGURE 1:**
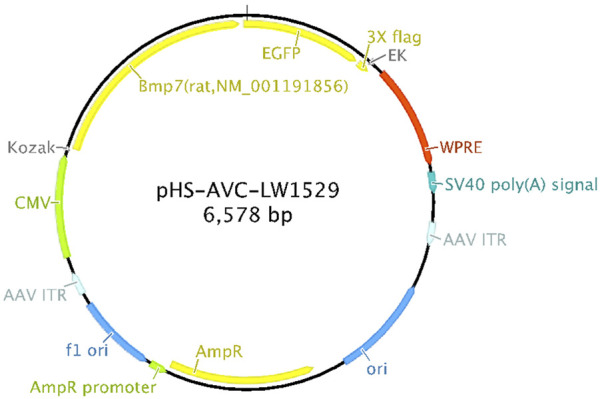
Schematic diagram of overexpressed adeno-associated virus backbone.

The *in vivo* overexpression of the BMP-7 sequence is shown in Table 1, and the schematic diagram of adeno-associated virus construction is shown in Figure 1.

**TABLE 1:** Virus sequence.

	Bmp7 (rat,NM_001191856)
pHS-AVC-LW1529	5′-ATG​CAC​GTG​CGC​TCG​CTG​CGC​GCT​GCG​GCG​CCA​CAC​AGC​TTC​GTG​GCG​CTC​TGG​GCG​CCT​CTG​TTC​TTG​CTG​CGC​TCT​GCC​TTG​GCC​GAC​TTC​AGC​CTG​GAC​AAC​GAG​GTG​CAC​TCC​AGT​TTC​ATC​CAC​CGG​CGC​CTC​CGC​AGT​CAG​GAG​CGG​CGG​GAG​ATG​CAG​CGG​GAA​ATC​CTG​TCC​ATC​TTG​GGC​TTG​CCC​CAT​CGT​CCG​CGC​CCG​CAC​CTC​CAG​GGA​AAA​CAT​AAT​TCG​GCG​CCC​ATG​TTC​ATG​TTG​GAC​CTG​TAC​AAC​GCC​ATG​GCG​GTG​GAG​GAG​AGT​GGG​CCG​GAC​GGA​CAG​GGC​TTC​TCC​TAC​CCC​TAC​AAG​GCC​GTC​TTC​AGT​ACC​CAG​GGT​CCC​CCT​TTG​GCC​AGC​CTG​CAG​GAC​AGC​CAC​TTC​CTC​ACC​GAC​GCC​GAC​ATG​GTC​ATG​AGC​TTC​GTC​AAC​CTA​GTG​GAG​CAC​GAC​AAG​GAA​TTC​TTC​CAC​CCT​CGA​TAC​CAC​CAT​CGA​GAG​TTC​CGG​TTT​GAT​CTT​TCC​AAG​ATC​CCC​GAG​GGA​GAG​GCG​GTG​ACC​GCA​GCC​GAG​TTC​AGG​ATC​TAT​AAG​GAC​TAC​ATC​CGG​GAG​CGG​TTT​GAC​AAC​GAG​ACC​TTC​CAG​ATC​ACA​GTC​TAT​CAG​GTG​CTC​CAG​GAG​CAC​TCA​GGC​AGG​GAG​TCC​GAC​CTC​TTC​TTG​CTG​GAC​AGC​CGT​ACC​ATC​TGG​GCT​TCT​GAG​GAG​GGC​TGG​TTG​GTA​TTT​GAC​ATC​ACA​GCC​ACC​AGC​AAC​CAC​TGG​GTG​GTC​AAC​CCT​CGG​CAC​AAC​CTG​GGC​TTA​CAG​CTC​TCC​GTG​GAG​ACC​CTG​GAT​GGG​CAG​AGC​ATC​AAC​CCC​AAG​TTG​GCA​GGC​CTG​ATT​GGA​CGG​CAT​GGA​CCC​CAG​AAC​AAG​CAA​CCC​TTC​ATG​GTG​GCC​TTC​TTC​AAG​GCC​ACG​GAG​GTT​CAT​CTC​CGT​AGC​ATC​CGG​TCC​ACG​GGG​GGC​AAA​CAA​CGC​AGC​CAG​AAC​CGC​TCC​AAG​ACT​CCA​AAG​AAC​CAA​GAG​GCA​CTG​AGG​ATG​GCC​AGT​GTG​GCA​GAA​AAC​AGC​AGC​AGT​GAC​CAG​AGG​CAG​GCC​TGC​AAG​AAA​CAC​GAG​CTG​TAT​GTT​AGC​TTC​CGA​GAC​CTT​GGC​TGG​CAG​GAC​TGG​ATC​ATC​GCA​CCT​GAA​GGC​TAT​GCT​GCC​TAC​TAC​TGT​GAG​GGA​GAG​TGT​GCC​TTC​CCT​CTG​AAC​TCC​TAC​ATG​AAC​GCC​ACC​AAC​CAT​GCT​ATC​GTC​CAG​ACA​CTG​GTT​CAC​TTC​ATC​AAC​CCA​GAC​ACC​GTA​CCC​AAG​CCC​TGC​TGT​GCC​CCC​ACC​CAG​CTC​AAC​GCG​ATA​TCT​GTC​CTC​TAC​TTC​GAC​GAC​AGC​TCC​AAC​GTC​ATC​CTG​AAG​AAG​TAC​AGA​AAC​ATG​GTG​GTC​CGG​GCC​TGT​GGC​TGC​CAC-3′

The experimental cells were NRK-52E cells (renal tubular epithelial cell line) derived from ATCC. Cell culture was performed using a glucose concentration of 5.5 mmol/L as the normal group and 30 mmol/L glucose concentration as the high glucose stimulation group, respectively. Westernblot experiments of cells were performed using the results of three batches of cells and counted.

### Reagents

The following reagents and instruments were used in the study: STZ (Sigma, United States). Total protein, creatinine, glucose, triglyceride, and cholesterol biochemical assay kits (Nanjing Jiancheng Company); immunohistochemistry SP two-step assay reagent (Beijing Zhongsun Jinqiao Biotechnology Co. Ltd.); Talent qPCR fluorescence quantification kit (Beijing Tiangen Biotechnology Co., Ltd.), BMP-7, FN, Id2, and GAPDH primers (Shanghai Biotechnology Engineering Service Co., Ltd.); low-sugar DMEM (5.5 mmol/L glucose) (Gibco Invitrogen, United States); fetal bovine serum (FBS) (Biological Industries, Israel); human recombinant BMP-7 (rhBMP-7; PeproTech); immunoprecipitation (Co-IP) kit (Thermo, United States); lipofectamine 2000 (Invitrogen, United States); rabbit anti-BMP-7 polyclonal antibody, rabbit anti-Vimentin polyclonal antibody, and rabbit anti-Col-III polyclonal antibody (Wuhan Proteintech Company); rabbit anti-α-SMA polyclonal antibody, rabbit anti-p38 polyclonal antibody, rabbit anti-p-p38 polyclonal antibody, rabbit anti-Erk1/2 antibody, and rabbit anti-p-Erk1/2 polyclonal antibody (Cell Signaling Technology); mouse anti-JNK monoclonal antibody, mouse anti-p-JNK monoclonal antibody (Santa Cruz, United States); mouse anti-Col-IV monoclonal antibody and rabbit anti-Id2 polyclonal antibody (Sigma, United States); rabbit anti-FN polyclonal antibody (Abcam); direct labeled secondary anti-GADPH-HRP antibody (Wuhan Ltd.); and SP600125 (JNK inhibitor), SB203580 (p38 inhibitor), and SCH772984 (Erk1/2 inhibitor) (Cell Signaling Technology).

### Histopathological Observation of Kidney Tissues

Kidney tissues were fixed in 4% paraformaldehyde and dehydrated using gradients of alcohol, half benzene and half alcohol, benzene, half benzene, and half wax; fixed by paraffin embedding and sectioned (3 μm) with a microtome; and the sections were used for HE staining, Masson staining, PAS staining, and immunohistochemical staining. Paraffin sections were baked at 60°C for 2 h; treated with xylene and gradient alcohol to chemical wax to water; stained using HE, Masson, and PAS staining kits; dehydrated; sealed with neutral resin; and observed microscopically. Similar methods were used for immunohistochemical staining with labeled antibodies.

### Western Blot Analysis for Protein Detection and PCR for RNA Detection

For analyses, 0.1 g of kidney tissue was added to 700 μl of lysate. 10% SDS-polyacrylamide gel was used to separate the protein, which was transferred to the PVDF membrane and blocked with skimmed milk. The membrane was intercepted according to different molecular weights and incubated with different primary antibodies. The p38, Erk, JNK, p-p38, p-Erk, and p-JNK antibodies (1:1000) were purchased from Cell Signaling Technology. BMP-7, Collagen III, and Vimentin (1:1000) were purchased from Proteintech. Collagen Ⅳ antibody (1:1000) was purchased from Sigma. FN antibody (1:1000) was purchased from Abcam and incubated overnight. The membrane was removed on the second day, washed three times with TBST (5 min/time), incubated with the secondary antibody (1:8000) for 2 h at room temperature, and exposed to Tanon apparatus.

Further, 0.1 g of tissue was lysed by adding 1 ml of TRIzol, and tissue RNA was extracted and reverse transcribed to obtain cDNA. Next, 20-μl Real-time PCR system (kit purchased from Tiangen) was prepared following the manufacturer’s protocol and detected using Bio-Rad PCR instrument. PCR was performed using calculated values for statistical analysis of the results, calculated by the formula 2^−ΔΔCt^.

PCR primer sequences are shown in Table 2.

**TABLE 2:** Primer sequences of RT-qPCR.

Primer	Primer sequences	Tm
BMP-7	F: CGT AGC ATC CGG TCC AC	55.0 (°C)
	R: CAG CTC GTG TTT CTT GCA G	
Fibronectin	F: ATT GCC TAC TCG CAG CTT	54.4 (°C)
	R: ACG GGA TCA CAC TTC CAC	
Id2	F: TGC TAC TCC AAG CTC AAG G	55.6 (°C)
	R: GTG TTC AGG GTG GTC AGC	
GADPH	F: GAC ATG CCG CCT GGA GAA AC	59.0 (°C)
	R: AGC CCA GGA TGC CCT TTA GT	

### Cell Experimental Grouping and Treatment

The cells requiring experimental treatment were inoculated in six-well plates at the time of passage. When the cells were in good growth condition and the fusion degree reached 70%, they were transferred to a low-sugar medium without FBS and synchronized for 12 h. The cells were divided into normal sugar (NG), high sugar (HG), and their additive/cytokine groups. The cell proteins were collected after 48 h, and cell immunofluorescence experiments were performed.

### Main Analysis Methods and Software

Data were statistically analyzed using SPSS25.0 and expressed as mean ± standard deviation. ANOVA was used for comparison between multiple groups of factors. Independent-samples *t* test was used for comparison between two groups, and differences were considered statistically significant at *p* ≤ 0.05. ImageJ 180 was used for image processing. Statistical software IBM SPSS statistics 19.0 was used for data processing. GraphPad Prism 5 was used to draw charts.

### Cell Immunofluorescence Staining

The cell suspension was inoculated into a 6-well plate/12-well plate lined with a cell crawl (the cell crawl was baked with an alcohol lamp, put into a six-well plate, and washed once with sterile saline). The suspension was transferred to a normal-sugar and a high-sugar medium when the fusion degree reached 50%, and removed after 48 h of incubation. The cells were washed three times with PBS, 5 min each time, fixed with 4% paraformaldehyde at room temperature for 20 min, and again washed three times with PBS, 5 min each time. They were treated with 0.5% Triton X-100 at room temperature for 20 min, and again washed three times with PBS, 5 min each time. For serum closure, the cells were aspirated with PBS, mixed with 10% BSA dropwise (BSA powder freshly prepared), and closed at 37°C for 30 min. The closure solution was washed off, primary antibodies were added dropwise, and the suspension was diluted with 1% BSA and refrigerated at 4°C overnight. The suspension was taken out from the refrigerator the next day for re-warming, the primary antibodies were recovered, and the cells were washed with PBS three times, 5 min each time. Secondary antibody incubation: CY3 sheep anti-mouse IgG (1:100) and FITC sheep anti-rabbit IgG (1:200) were added dropwise, and the cells were incubated at 37°C for 1 h in the absence of light and washed three times with PBS, 5 min each time. DAPI was added dropwise to re-stain nuclei, and the cells were incubated for 5 min in the absence of light and washed four times with PBS, 5 min each time. The excess liquid was absorbed from the crawling film with an absorbent paper, and the film was sealed with a sealer containing a fluorescence quencher and observed under a fluorescence microscope.

## Results and Conclusion

### Changes in Biochemical Indices and Pathological Staining in Rats

As shown in Table 3, the blood glucose level was significantly higher in the DM group compared with the NC group. The blood glucose in the DM rats decreased after OMT treatment but was still higher than normal, suggesting that OMT could interfere with the blood glucose level *in vivo* but did not achieve an effective therapeutic effect. The blood glucose level did not change significantly after the injection of BMP-7 adeno-associated virus, suggesting that BMP-7 did not affect the process of DKD by regulating the blood glucose level. The levels of triglycerides and total cholesterol increased in the DM group and decreased after OMT treatment, but did not significantly improve in the rAAV + BMP-7 group, suggesting that OMT had a therapeutic effect on lipid metabolism in early DKD. In contrast, BMP-7 did not have a significant effect on lipid metabolism in DKD. The 24-h urine protein quantification reflected that DM rats showed some renal damage compared with that in the NC group. The 24-h urine protein quantification reflected that DM rats showed some renal injury, which was improved by OMT. It also improved in the rAAV + BMP-7 group, suggesting that BMP-7 played a role in promoting the improvement in renal injury in early DKD.

**TABLE 3:** Detection of renal function and metabolism-related indicators in each group.

Group	NC	DM	OMT	rAAV + BMP-7
Glucose (mmol/L)	5.54 ± 1.12	36.75 ± 4.46*	23.43 ± 4.77#	34.49 ± 4.43
Triglyceride (mmol/L)	0.75 ± 0.10	2.24 ± 1.23*	0.74 ± 0.19#	1.90 ± 1.17
Cholesterol (mmol/L)	1.38 ± 0.24	2.58 ± 0.36*	1.60 ± 0.17#	2.55 ± 0.17
Urine creatinine (μmol/L)	8581.85 ± 2755.24	748.42 ± 244.37*	1151.42 ± 137.67#	1216.42 ± 143.98#
Serum creatinine (μmol/L)	15.78 ± 3.52	150.62 ± 55.10*	21.04 ± 16.68#	28.28 ± 12.07#
Creatinine clearance rate (ml/min)	8.71 ± 3.34	0.64 ± 0.26*	6.46 ± 3.92#	5.95 ± 2.80#
24 h urinay protein quantity (mg)	13.42 ± 3.27	47.74 ± 14.74*	26.95 ± 10.30#	31.52 ± 8.71#

*p < 0.05 vs. NC; #*p* < 0.05vs. DM; Ccr (ml/min) = urinary creatinine(μmol/l) × urine volume(ml/min)/blood creatinine(μmol/l).

**FIGURE 2:**
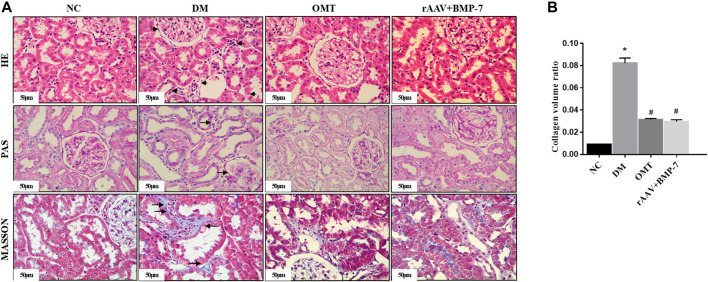
Paraffin sections of rat kidney tissues in each group were stained with HE, PAS, and MASSON(scale bar is 50 μm), and the ratio of collagen area to the total area of the tissue under each field was counted, ^*^ *p* < 0.05 vs. NC group, ^#^ *p* < 0.05 vs. DM group.

HE staining and PAS staining showed that the glomerular lamina propria cells in the NC group did not show significant hyperplasia, the thylakoid region did not show significant widening, the basement membrane did not show significant thickening, the renal tubules were tightly arranged and did not show atrophy, the epithelial cells did not show significant granular and vacuolar degeneration, the renal interstitium did not show abnormalities, and the small vessels did not show glassy degeneration and thickening (Figures 2A,B). The DM group showed mild segmental hyperplasia of thylakoid cells and stroma, no significant thickening of basement membrane, small focal atrophy of renal tubules (atrophy area less than 10%), granular and vacuolar degeneration of epithelial cells in focal areas, small focal lymphocytic infiltration and fibrous tissue hyperplasia in the renal interstitium (shown by arrows in the figure), and no glassy degeneration and significant thickening of small vessel walls. In the OMT group and the rAAV + BMP-7 intervention group, there was a reduction in the area of small focal tubular atrophy compared with the DM group, an improvement in microscopic epithelial cell morphology, less lymphocytic infiltration in the renal interstitium, and a reduction in the area of fibrous tissue hyperplasia. The results of Masson staining and statistical analysis of collagen volume showed that the blue collagen deposition in the tubular interstitium significantly increased in the DM group compared with the NC group. The OMT and rAAV + BMP-7 intervention groups showed some collagen distribution and reduced fibrosis. The results suggested that the overexpression of BMP-7 and OMT treatment improved renal injury and inhibited the development of fibrosis.

### BMP-7 Ameliorated Tubulointerstitial Fibrosis in DKD

**FIGURE 3:**
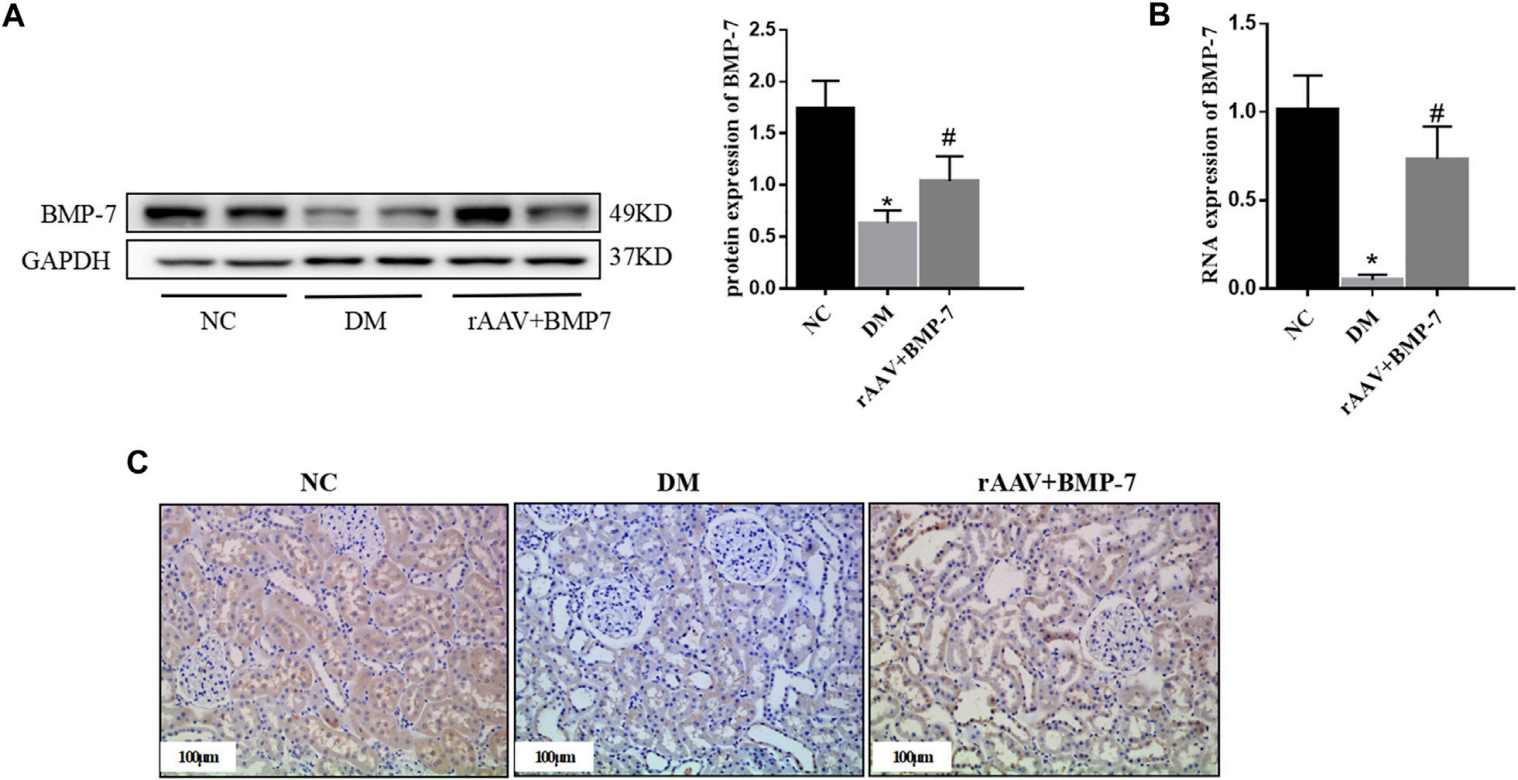
To verify the changes of BMP-7 in tissue after overexpression of BMP-7 *in vivo*. **(A)** Western blot was used to detect BMP-7 protein expression levels in the normal control group, diabetes model group, and rAAV + BMP-7 group. **(B)** Real-time PCR was used to detect tissue BMP-7 RNA expression levels. ^*^ *p* < 0.05 vs. NC group, ^#^ *p* < 0.05 vs. DM group. **(C)** Immunohistochemistry was used to detect the expression of BMP-7 in the paraffin sections of kidney tissue in the normal control group, the diabetes model group, and the rAAV + BMP-7 group (scale bar is 100 μm).

Adeno-associated viruses carrying the overexpressed BMP-7 gene were constructed and then injected into the tail vein 6 weeks after DM modeling. The results showed that the protein and RNA expression levels of BMP-7 decreased in the DM group, while BMP-7 protein (Figure 3A) and RNA (Figure 3B) expression levels significantly increased in the rAAV + BMP-7 group compared with the DM group. The immunohistochemical results (Figure 3C) showed that the expression of BMP-7 significantly decreased in the kidneys of rats in the DM group; the expression increased after the injection of the overexpressed adeno-associated virus compared with that in the DM group. The results suggested that the expression of BMP-7 decreased under the high-glucose condition. In contrast, the expression level of BMP-7 was significantly higher in the rAAV + BMP-7 group than in the DM group, and the model was successfully replicated.

**FIGURE 4:**
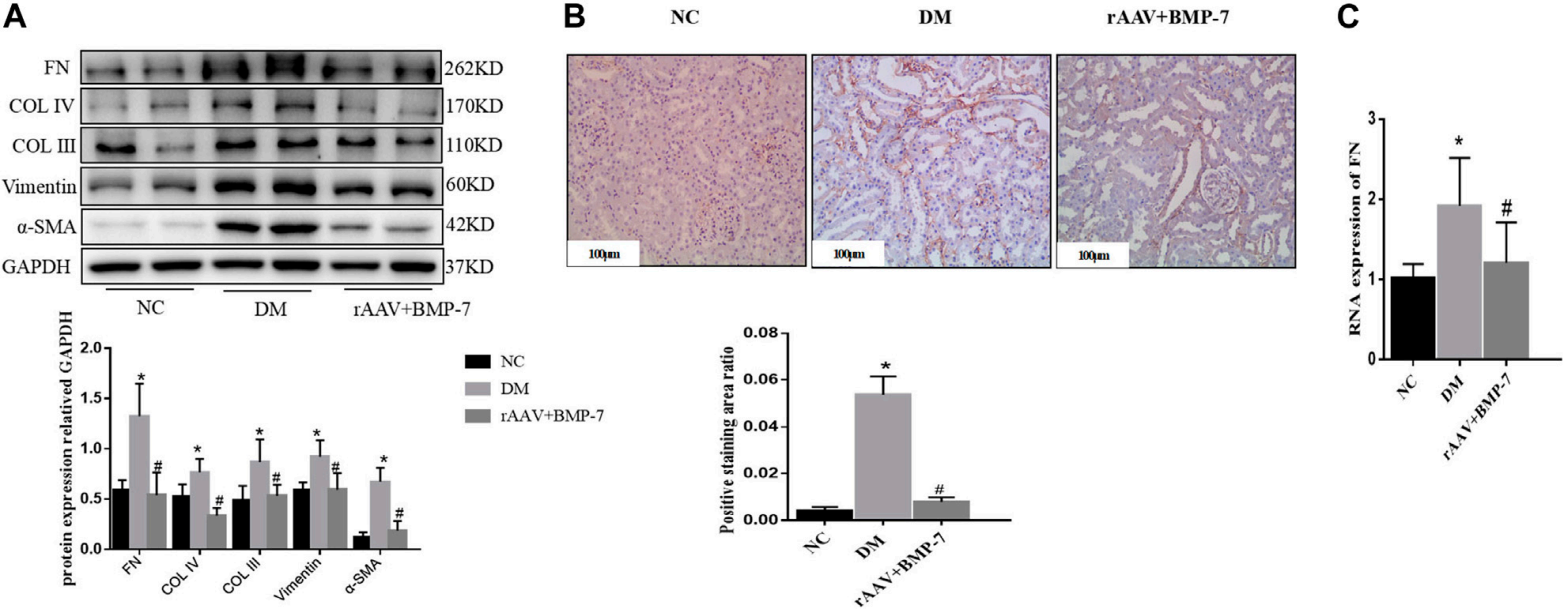
Detection of fibrosis-related index levels after overexpression of BMP-7 *in vivo*. **(A)** Western blot was used to detect EMT and ECM related indexes in the normal control group, diabetes model group, and rAAV + BMP-7 group, *p < 0.05 vs. NC group, #*p* < 0.05 vs. DM group. **(B)** Immunohistochemistry was used to detect the expression of FN in the paraffin sections of kidney tissue in the normal control group, the diabetes model group, and the rAAV + BMP-7 group, and the ratio of the positive staining area to the total tissue area under each field was counted. *p < 0.05 vs. NC group, #*p* < 0.05 vs. DM group (scale bar is 100 μm) **(C)** Real-time PCR was used to detect tissue FN RNA expression levels. *p < 0.05 vs. NC group, #*p* < 0.05 vs. DM group.

Western blot analysis results showed that the expression of Vimentin and α-SMA increased in the DM group compared with the NC group, suggesting that EMT occurred in the DM group, while the expression of Col III, Col IV, and FN increased, suggesting that ECM deposition in the kidney tissue increased in the DM group (Figure 4A). The levels of the aforementioned indicators decreased In the rAAV + BMP-7 group, suggesting that the expression of BMP-7 was upregulated after the tail vein injection of BMP-7 adeno-associated virus, which could promote the improvement in the EMT process and decrease in ECM deposition. Immunohistochemistry showed an increase in FN deposition in the interstitial kidney tissue in the DM group compared with the NC group and a decrease in the rAAV + BMP-7 group compared with the DM group (Figure 4B). Real-time PCR showed a decrease in the RNA level of FN in the rAAV + BMP-7 group compared with the DM group (Figure 4C). These results suggested that the restoration of BMP-7 expression could inhibit the fibrosis-related process in DM rats.

**FIGURE 5:**
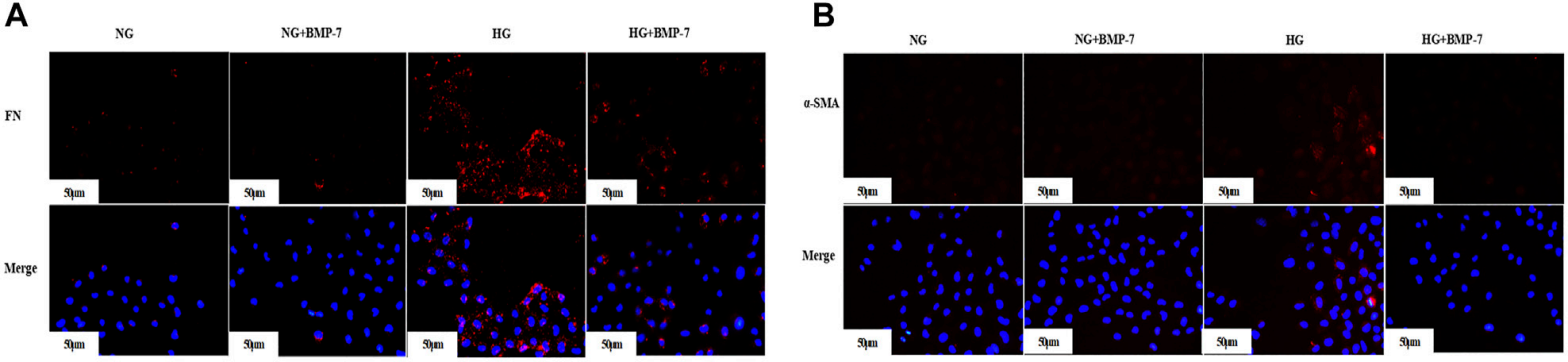
**(A)** The expression of FN in cells was detected by immunofluorescence (scale bar is 50 μm). **(B)** The expression of α-SMA in cells was detected by immunofluorescence (scale bar is 50 μm).

Pre-laboratory studies confirmed that BMP-7 improved the EMT- and ECM-related indexes in high glucose–stimulated NRK-52E cells. The expression of FN (Figure 5A) and α-SMA (Figure 5B) in NRK-52E cells was detected by cellular immunofluorescence. The results verified that the expression of FN and α-SMA reduced after the administration of BMP-7 cytokine compared with that in the HG group. These results suggested that *in vitro* BMP-7 delayed the EMT process in renal tubular epithelial cells under the high-glucose status.

### BMP-7 Upregulated Id2 Expression and its Possible Signaling Pathway

**FIGURE 6:**
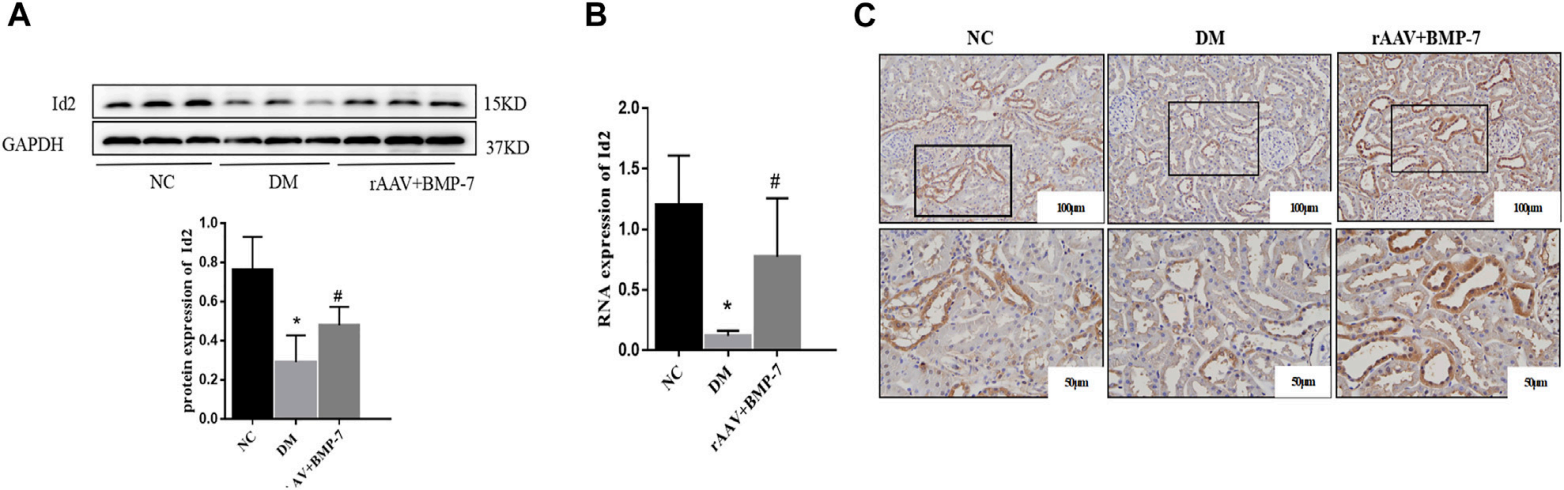
To verify the changes in protein and mRNA levels of Id2 after overexpression of BMP-7 *in vivo*. **(A)** Western blot was used to detect Id2 protein expression levels in the normal control group, diabetes model group, and rAAV + BMP-7 group, *p < 0.05 vs. NC group, #*p* < 0.05 vs. DM group. **(B)** Real-time PCR was used to detect tissue Id2 RNA expression levels. *p < 0.05 vs. NC group, #*p* < 0.05 vs. DM group. **(C)** Immunohistochemistry was used to detect the expression of Id2 in the paraffin sections of kidney tissue in the normal control group, the diabetes model group, and the rAAV + BMP-7 group (scale bar are 100 and 50 μm).

Western blot analysis showed that the protein expression level of Id2 in the DM group significantly decreased in the kidney tissues, and the protein level of Id2 increased after the overexpression of BMP-7 (Figure 6A). Fluorescence quantitative PCR results showed that the RNA expression of Id2 reduced in the DM group compared with the NC group (Figure 6B). At the same time, its RNA expression level was upregulated after the overexpression of BMP-7. The immunohistochemical results showed that Id2 was abundantly expressed in the cytoplasm and nucleus of renal tubular epithelial cells in the NC group, while it was significantly reduced in the DM group and increased in the rAAV + BMP-7 group compared with the DM group (Figure 6C). The results suggested that Id2 expression was reduced in the high-glucose status and upregulated by the overexpression of BMP-7.

**FIGURE 7:**
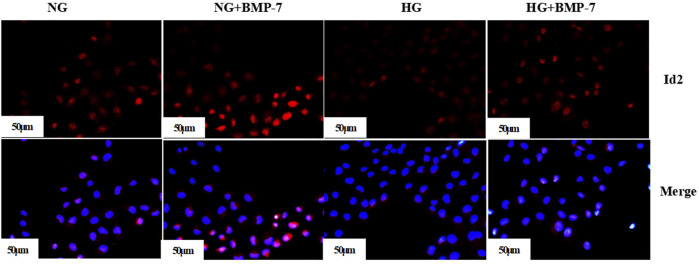
The expression of Id2 in cells was detected by immunofluorescence (scale bar is 50 μm).

Pre-laboratory studies confirmed that the addition of BMP-7 to NRK-52E cells cultured in standard- and high-glucose medium also upregulated Id2 expression. The cellular immunofluorescence results showed that Id2 expression in HG-cultured NRK-52E cells was significantly lower compared with that in the NG group, and Id2 expression increased after the addition of BMP-7 cytokine treatment (Figure 7). The results verified that BMP-7 could upregulate Id2 expression in NRK-52E cells.

**FIGURE 8:**
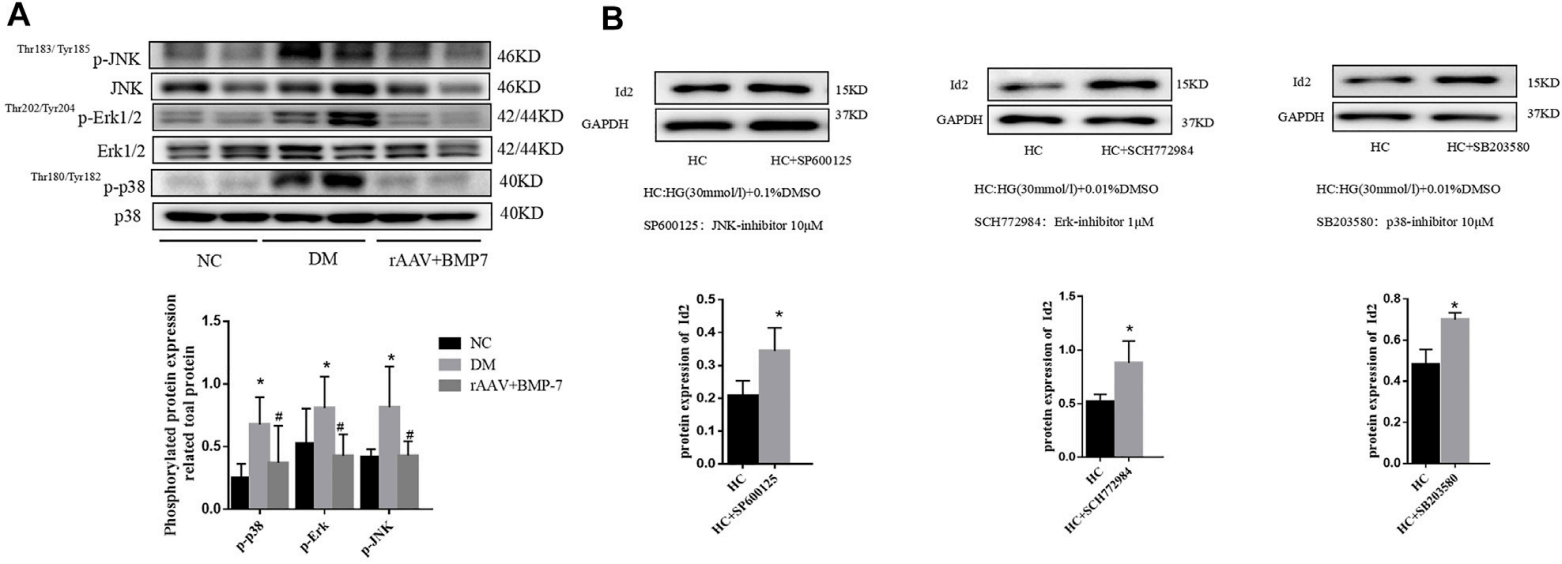
**(A)** Western blot was used to detect the phosphorylation of p38, Erk1/2, and JNK in the normal control group, diabetes model group, rAAV + BMP-7 group, ^*^ *p* < 0.05 vs. NC group, ^#^ *p* < 0.05 vs. DM group. **(B)** The expression of Id2 in cells was detected by Western blot, ^*^ *p* < 0.05 vs. HC group.

The Western blot analysis showed that the phosphorylation levels of p38, Erk1/2, and JNK significantly increased in renal tissues in the DM group, and the activation of p38, Erk1/2, and JNK was significantly inhibited after the overexpression of BMP-7 (Figure 8A). The results suggested that the overexpression of BMP-7 could inhibit the activation of the nonclassical MAPK pathway.

Three inhibitors of MAPK family, SP600125 (JNK inhibitor), SB203580 (p38 inhibitor), and SCH772984 (Erk1/2 inhibitor), were added to NRK-52E cells for 2 h and then transferred to serum-containing high-glucose medium for 48 h. The cell proteins were extracted, and Western blot analysis was performed to detect the change in Id2 expression. The results showed that the protein expression level of Id2 was upregulated after the addition of the three inhibitors compared with that in the DMSO-stimulated control (HC) group (Figure 8B). The results suggested that the expression of Id2 increased when the activation of the MAPK (p38, Erk1/2, and JNK) signaling pathway was inhibited.

### OMT Affected the BMP-7–MAPK Signaling Pathway to Improve Fibrosis

**FIGURE 9:**
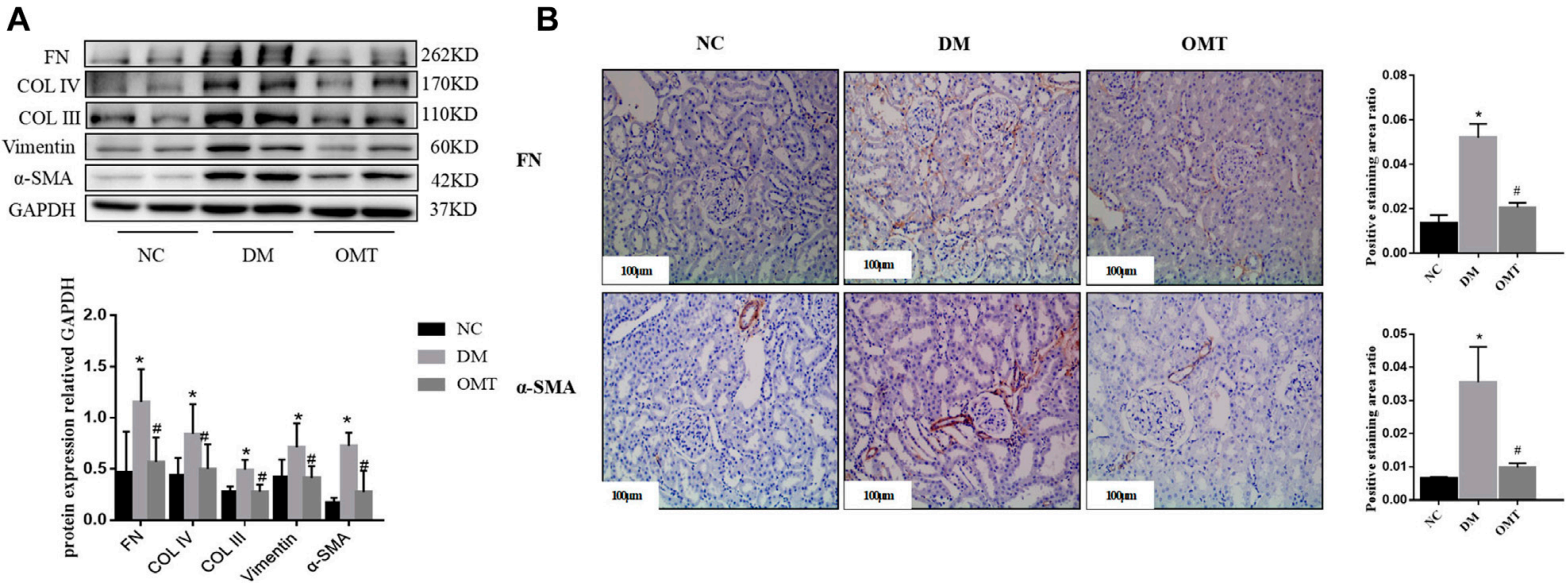
Detection of fibrosis changes after OMT treatment *in vivo*. **(A)** Changes in the expression of EMT and ECM-related indicators in the normal control group, diabetes model group, OMT intervention group, ^*^ *p* < 0.05 vs. NC group, ^#^ *p* < 0.05 vs. DM group **(B)** Immunohistochemical technology was used to detect the expression of FN and α-SMA in paraffin sections of rat kidney tissues in each group (scale bar is 100 μm), and the ratio of the positive staining area to the total area of the tissue under each field was counted, ^*^ *p* < 0.05 vs. NC group, ^#^ *p* < 0.05 vs. DM group.

Western blot results showed that the protein expression levels of FN, Col IV, and Col III decreased in DM rats after OMT intervention compared with that in the DM group, suggesting a decrease in ECM deposition (Figure 9A). The expression of Vimentin and α-SMA decreased, suggesting that the EMT process was slowed down and the degree of fibrosis was improved. Immunohistochemical results showed that FN deposition reduced and α-SMA expression relatively decreased in the renal tissues of DM rats after OMT treatment (Figure 9B). These results suggested that oxymatrine has a significant ameliorative effect on DKD kidney injury.

**FIGURE 10:**
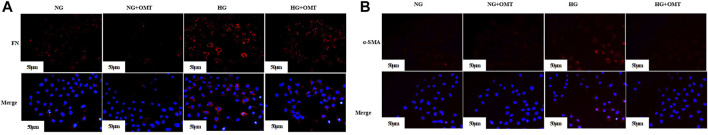
**(A)** The expression of FN in cells was detected by immunofluorescence (scale bar is 50 μm). **(B)** The expression of α-SMA in cells was detected by immunofluorescence (scale bar is 50 μm).

Pre-laboratory studies confirmed that OMT improved the EMT- and ECM-related indexes in high glucose–stimulated NRK-52E cells. The expression of FN (Figure 10A) and α-SMA (Figure 10B) in NRK-52E cells was detected by cellular immunofluorescence. The results showed a relative reduction in FN and α-SMA expression in high-glucose-stimulated NRK-52E cells after OMT intervention. The results verified that OMT could reduce ECM deposition in high-glucose-stimulated NRK-52E cells.

**FIGURE 11:**
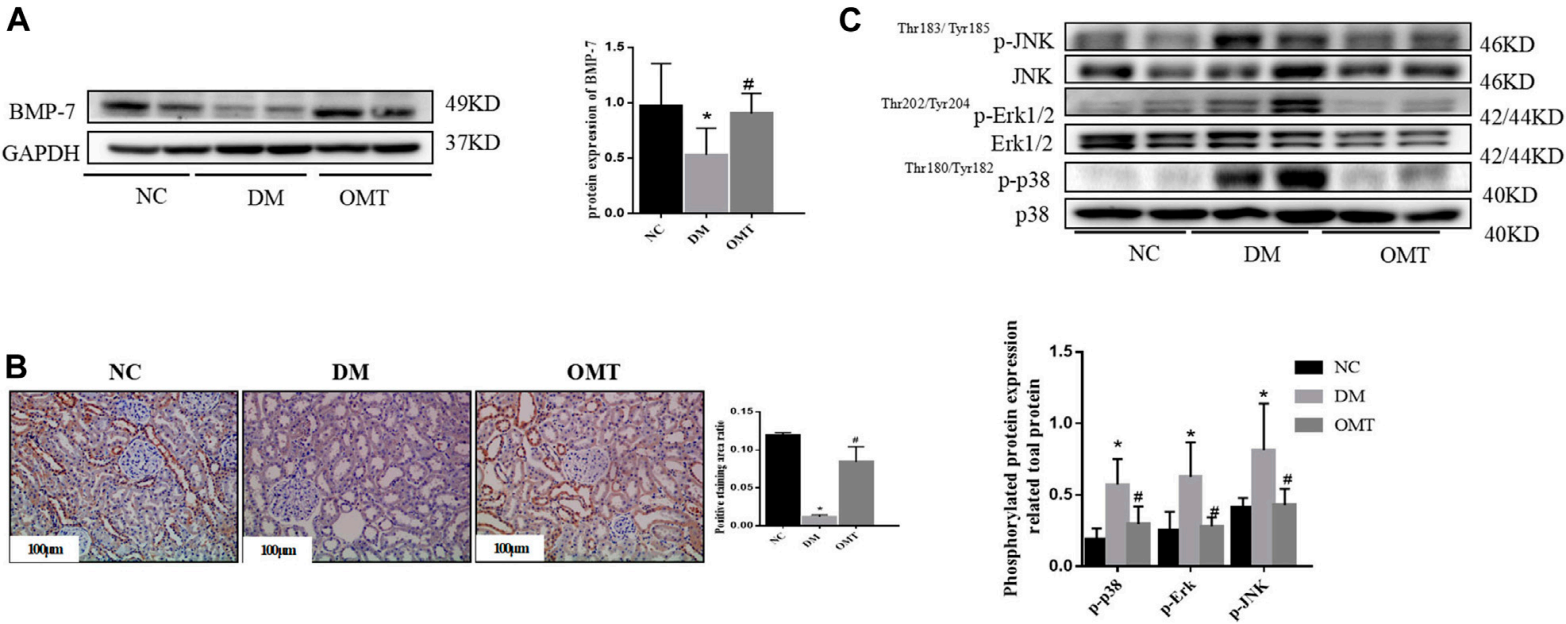
**(A)** Detection of BMP-7 proteins after OMT intervention. Western blot was used to detect the change of BMP-7 expression level after OMT intervention, *p < 0.05 vs. NC group, #*p* < 0.05 vs. DM group. **(B)** The immunohistochemical technique was used to detect the expression of BMP-7 in the paraffin sections of rat kidney tissues in each group (scale bar is 100 μm), and the ratio of the positive staining area to the total area of the tissue under each field was counted, *p < 0.05 vs. NC group, #*p* < 0.05 vs. DM Group **(C)** Detection of MAPK pathway-related proteins after OMT intervention. Western blot was used to detectp38, Erk and JNK phosphorylation levels after OMT intervention, *p < 0.05 vs. NC group, #*p* < 0.05 vs. DM group.

OMT has a certain ameliorative effect on renal injury in early DKD, but its exact mechanism of action is not fully understood. The Western blot analysis showed that OMT treatment increased the protein expression level of BMP-7 (Figure 11A). Immunohistochemistry showed that BMP-7 expression significantly increased in the kidneys after OMT intervention (Figure 11B). Meanwhile, the phosphorylation levels of MAPK p38, Erk1/2, and JNK were inhibited after OMT treatment (Figure 11C). The results suggested that OMT could upregulate the expression of BMP-7 in DKD kidney tissues and then inhibited the activation of MAPKs.

## Discussion

DKD can be defined as persistent microalbuminuria and impaired glomerular filtration leading to the deterioration of renal function and ultimately morbidity and mortality in patients. Proteinuria is a characteristic clinical biomarker of DKD in patients with diabetes (Danta et al., 2021). In this study, STZ was used to partially destroy the pancreatic β-cells of SD rats to replicate the type 1 diabetes model. The results of biochemical-related indexes showed elevated blood glucose levels and significantly increased expression levels of triglycerides and total cholesterol in the DM group compared with the NC group, suggesting successful replication of the diabetes model. The 24-h urine protein quantification significantly increased, suggesting early renal injury in the DM group.

The main manifestations of early renal pathology in DKD include proliferation and hypertrophy of renal cells, thickening of the glomerular basement membrane, and expansion of the ECM (Akhtar et al., 2020); they are associated with the EMT process. This process leads to massive myofibroblast activation and proliferation. It increases ECM secretion through pro-fibrogenic factors. Increased ECM cannot be degraded rapidly, leading to excessive accumulation of glomerular and tubulointerstitial ECM and causes renal functional impairment. The pathomorphological staining showed mild segmental hyperplasia of thylakoid cells and stroma, tubular atrophy, and interstitial fibrous tissue hyperplasia in the DM group, suggesting glomerular and tubulointerstitial fibrotic lesions in the kidney. Western blot results showed that the expression of mesenchymal cell markers α-SMA and Vimentin significantly increased in the DM group compared with the NC group rats, suggesting a significant increase in the expression of FN, Col IV, and Col III and the presence of excessive ECM deposition. These results indicated that the rats in the DM group had early kidney injury.

Recent studies have confirmed the ability of Id2 to resist organ fibrosis by inhibiting the EMT process (Saika et al., 2006; Jibing et al., 2015). Veerasamy et al., (2013) showed that Id2 reduction was essential for TGF-β-induced α-SMA expression in human proximal tubular epithelial cells. Pre-laboratory studies showed that the downstream-related protein expression of EMT could be inhibited by upregulating Id2 expression. The results of the present study further confirmed that the Id2 expression level in the kidneys was reduced in the DM group compared with the NC group. However, the specific targets of Id2 regulation of EMT-related proteins remain to be clarified. As a critical transcription factor regulating EMT, Snail is involved in the EMT process in DKD (Bai et al., 2016). In the OSCC model, Id2 could bind to Snail and thus regulate the EMT process (JingPing et al., 2015; Kamata et al., 2016; Sumida et al., 2016). Id2 bound to the SNAG domain of Snai1 at the β4 promoter in normal murine mammary gland cells, and hence the function of Snai1 was inhibited (Cheng et al., 2013). In this study, the Western blot analysis (Supplementary Figure S1A) after the transfection of cells with Id2 siRNA showed that the protein expression level of Snail remained unchanged after the knockdown of Id2. The results of Co-IP (Supplementary Figure S1B) and immunofluorescence double staining (Supplementary Figure S1C) showed that Id2 bound and co-localized with Snail, which again verified that Id2 did not affect Snail expression.

Under physiological conditions, some positive regulators of fibrogenesis are tightly controlled by negative regulators, and the expression of these negative regulators is often reduced in organ tissues with fibrotic lesions. It may be an essential factor contributing to the development of fibrosis. Domestic and foreign scholars demonstrated that BMP-7 was an antifibrotic cytokine with a significant antifibrotic effect (Sun et al., 2017; Song et al., 2020; Kong et al., 2021; Mo et al., 2021). In this study, Western blot results showed that the expression level of BMP-7 was significantly lower in the DM group rats than in the NC group rats. The immunohistochemical results showed that the expression of BMP-7 decreased in the kidney tissues of the DM group rats. As a negative regulation−related protein of DKD, BMP-7 cytokine treatment in NRK-52E cells significantly improved the EMT- and ECM-related indexes (Xiao et al., 2019). In addition, we used adeno-associated virus as a vector to construct an animal model overexpressing BMP-7. The results showed that the expression levels of Col III, Col IV, and FN, the fibrosis-related indexes in kidney tissues, were downregulated after the overexpression of BMP-7 compared with those in the DM group. The 24-h urine protein quantification of the kidney function−related indexes significantly decreased but the metabolism-related indexes were not significantly changed. The morphological staining suggested that the degree of interstitial fibrosis reduced after the overexpression of BMP-7, which confirmed the protective effect of BMP-7 on renal injury in DM rats. In addition, BMP-7 cytokine intervention was given to NRK-52E cells cultured *in vitro* with a high glucose concentration; the expression of ECM-related proteins reduced after the addition of BMP-7. These results suggested that BMP-7 improved tubulointerstitial fibrosis by delaying the EMT process; however, the specific regulatory mechanism of BMP-7 on fibrosis remains unclear.

The MAPK family plays essential roles in a variety of signaling pathways, including inflammation, oxidative stress, and apoptosis. Excessive activation of the MAPK pathway promotes the EMT process in renal tubular epithelial cells, suggesting that the MAPK pathway is involved in the progression of early DKD (Geng et al., 2020). In the present study, Western blot results showed that the phosphorylation levels of P38, Erk1/2, and JNK significantly increased in the DM group, indicating that the MAPK pathway (p38, Erk1/2, and JNK) was in an activated state in the chronic kidney injury model in diabetic rats. One study found that BMP-7 treatment given to db/db mice inhibited tubular inflammatory responses by suppressing p38 and Erk1/2 signaling pathways (Li et al., 2015). *In vitro*, BMP-7 ameliorated glucose-induced oxidative stress by inhibiting JNK phosphorylation (Ching-Hua et al., 2009). The results of this study showed that the levels of MAPKs p38, Erk1/2, and JNK phosphorylation significantly decreased after the overexpression of BMP-7 *in vivo*, suggesting that BMP-7 could delay the EMT process and ECM deposition by inhibiting the activation of MAPKs under the high-glucose status, and thus improved fibrosis.

Another study found that BMP-7 increased Id2 expression to exert antifibrotic effects in organ fibrosis models (Kim et al., 2015). The results of the present study showed that the expression level of Id2 increased after the overexpression of BMP-7 in DM rats, suggesting that BMP-7 could improve organ fibrosis by upregulating the expression of Id2. We cultured NRK-52E renal tubular epithelial cells *in vitro* and administered three MAPK phosphorylation inhibitors separately after high-glucose stimulation for 48 h to further investigate the regulatory mechanism of BMP-7 on Id2. The results showed that the expression level of Id2 was elevated after the addition of MAPK inhibitors. The *in vitro* and *in vivo* results suggested that BMP-7 might upregulate Id2 expression by inhibiting the phosphorylation of the MAPK pathway.

Herbal medicine has potential clinical benefits as primary or alternative therapy for treating diabetic neuropathy due to its multi-targeted function. A large number of studies have emphasized the molecular mechanism of bioactive compounds of traditional Chinese medicine and Raynaud’s protective effect, which are involved in the signaling pathways of glucose/lipid metabolism regulation and exert antioxidant, anti-inflammatory, anti-fibrosis, and podocyte-protective effects (Tang et al., 2021). OMT has been shown to play a therapeutic role in visceral fibrosis, the exact mechanism of which is still poorly understood. The results of the present study showed that the 24-h urine protein quantification of renal function-related indexes and metabolic indexes, including glucose, triglycerides, and cholesterol, improved to different degrees, and pathomorphological staining showed a relative improvement in renal fibrotic lesions after the administration of OMT intraperitoneally to DM rats, confirming the therapeutic effect of OMT on renal injury in rats with early DKD. Western blot results showed that the expression Vimentin and α-SMA was downregulated compared with that in the DM group. It suggested that the EMT process was slowed down in the OMT group. The expression of FN, Col V, and Col III decreased, suggesting that ECM deposition was relatively reduced, which confirmed that the intervention of OMT improved the renal fibrosis process. Another study verified that the levels of p38, Erk1/2, and JNK phosphorylation were inhibited after the *in vitro* intervention of OMT in microglia (Dong et al., 2019). In the present study, the levels of p38, Erk1/2, and JNK phosphorylation were downregulated in the kidneys of rats in the OMT group compared with the DM group, suggesting that oxymatrine might regulate the EMT process in DKD kidneys by inhibiting MAPK phosphorylation levels. Further study showed that the expression level of BMP-7 in rat kidney tissues increased after OMT treatment. The protein expression level detected by Western blot was consistent with the immunohistochemical results of kidney tissues, suggesting that OMT could upregulate the expression level of BMP-7 *in vivo*. All these results suggested that OMT affected the BMP-7–MAPK pathway and thus improved the development of renal fibrosis in DKD.

In summary, OMT affected the expression of BMP-7 and then inhibited the phosphorylation level of MAPKs, the nonclassical pathway of BMP-7. Consequently, the expression of Id2 was upregulated, which inhibited the transcriptional activation of downstream target genes by binding to the EMT-related transcription factor Snail. This delayed the EMT process and ECM deposition in diabetic kidney fibrosis and improved the renal injury in early DKD, thus providing an experimental basis for clinical treatment and pharmacological intervention in early DKD.

## Data Availability

The original contributions presented in the study are included in the article/Supplementary Material, further inquiries can be directed to the corresponding authors.
