# Supplementary material for: BMP-7 Upregulates Id2 Through the MAPK Signaling Pathway to Improve Diabetic Tubulointerstitial Fibrosis and the Intervention of Oxymatrine
Source: Front Pharmacol. 2022 Jun 2;13:900346. doi: 10.3389/fphar.2022.900346 (PMC9201778; doi:10.3389/fphar.2022.900346)
Supplement: Supplementary file 1 [file DataSheet1.docx]

Supplementary Material


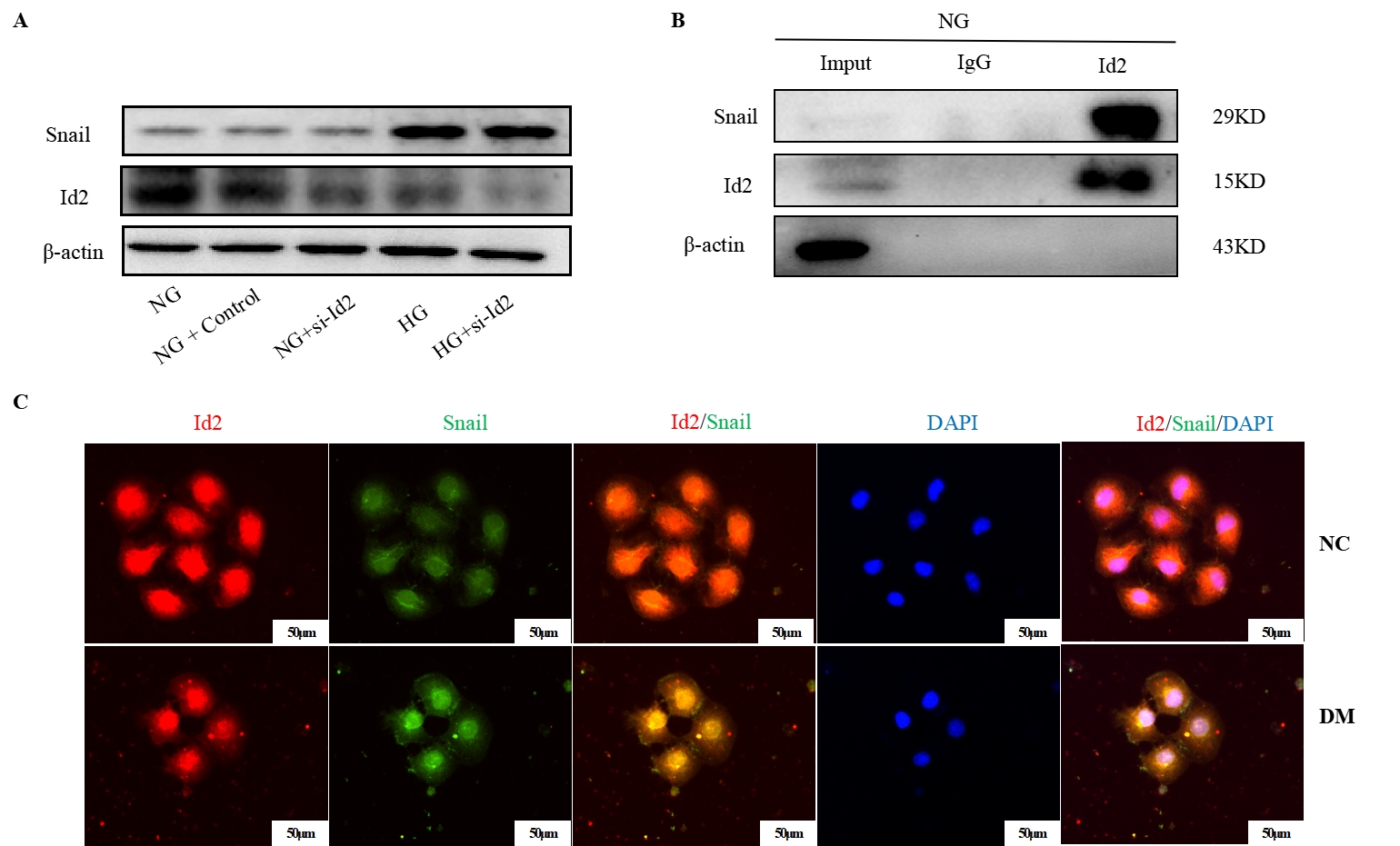


**Supplementary Figure 1.** (A)Knock down Id2 in NRK-52E cells, and western blot verifies protein expression changes of Snail. (B)Combined localization verification of Id2 and Snail in NRK-52E cell by CO-IP . (C)Combined localization verification of Id2 and Snail in NRK-52E cell by immunofluorescence (scale bar is 50μm).
